# A Commentary on Electrographic Seizure Management and Clinical Outcomes in Critically Ill Children

**DOI:** 10.3390/children10020258

**Published:** 2023-01-31

**Authors:** Lily Tran, Rebecca Welcher, Rodney Scott

**Affiliations:** Nemours Children’s Health System Division of Neurology, 1600 Rockland Rd., Wilmington, DE 19803, USA

**Keywords:** electrographic seizures, outcome, critically Ill children

## Abstract

Continuous EEG (cEEG) monitoring is the gold standard for detecting electrographic seizures in critically ill children and the current consensus-based guidelines recommend urgent cEEG to detect electrographic seizures that would otherwise be undetected. The detection of seizures usually leads to the use of antiseizure medications, even though current evidence that treatment leads to important improvements in outcomes is limited, raising the question of whether the current strategies need re-evaluation. There is emerging evidence indicating that the presence of electrographic seizures is not associated with unfavorable neurological outcome, and thus treatment is unlikely to alter the outcomes in these children. However, a high seizure burden and electrographic status epilepticus is associated with unfavorable outcome and the treatment of status epilepticus is currently warranted. Ultimately, outcomes are more likely a function of etiology than of a direct effect of the seizures themselves. We suggest re-examining our current consensus toward aggressive treatment to abolish all electrographic seizures and recommend a tailored approach where therapeutic interventions are indicated when seizure burden breaches above a critical threshold that may be associated with adverse outcomes. Future studies should explicitly evaluate whether there is a positive impact of treating electrographic seizures or electrographic status epilepticus in order to justify continuing current approaches.

## 1. Introduction

**Figure 1:**
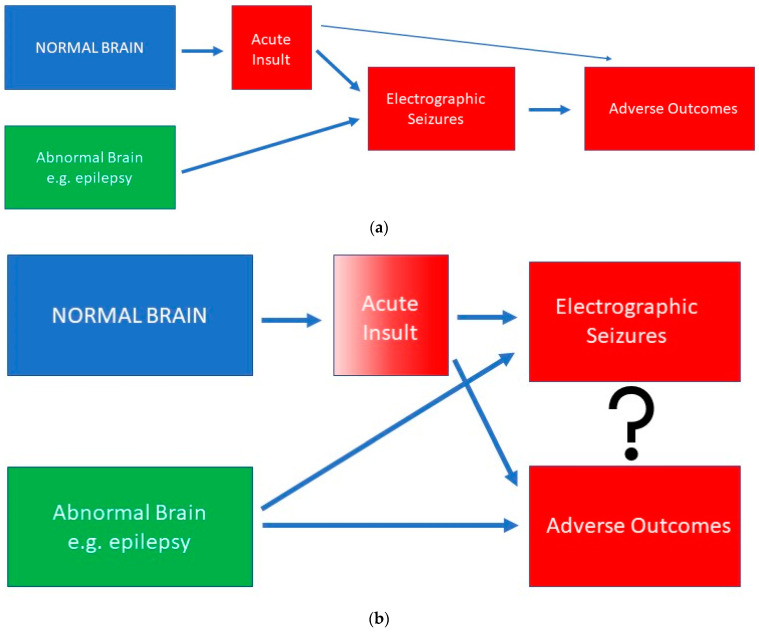
Conceptual framework for the relationships between electrographic seizures and adverse outcomes. (**a**) shows a causative relationship where the electrographic seizures *per se* make a significant contribution to outcomes. There is an impact of the underlying etiology, particularly if there is an acute neurological insult, but this currently receives less attention than the potential impact of electrographic seizures. (**b**) shows a correlation where the etiology is the main predictor of outcomes. As the severity of the insult increases, the likelihood that electrographic seizures will occur increases and the likelihood of an adverse outcome increases. The impact of electrographic seizures on the outcomes is likely small and only in the context of electrographic status epilepticus.

It is widely accepted that children with critical illnesses that are admitted to pediatric intensive care units may have epileptic abnormalities in their EEG [1,2,3,4,5]. The identification of such abnormalities often results in treatment with antiseizure medications [6], with the implicit expectation that this will improve their neurological outcomes. However, recent studies and the re-evaluation of previous studies raises a correlation versus causation concern that we will discuss in this commentary (Figure 1).

Continuous-EEG monitoring (cEEG) detects electrographic seizures in 10–40% of children in the pediatric intensive care unit (PICU) or emergency department [2,7]. This observation resulted in the use of cEEG in PICU increasing by 30% between 2010 and 2011 [7] and drove the development of clinical guidelines for the use of cEEG [8]. These consensus based guidelines strongly recommend that cEEG should be widely used and that these abnormalities should be treated [8]. The guidelines were successfully implemented with increased adherence to cEEG after the publication of guidelines [9]. Broad acceptance of the guidance has led to increased detection of electrographic seizures with downstream changes in clinical management, mostly in the form of new drugs or increased doses of the current drug when electrographic seizures are identified [3]. In a survey of adult and child neurologists, 63% aimed to obliterate all electrographic seizures and 5% would treat only if electrographic status epilepticus was detected [3]. Thus, the idea that electrographic abnormalities in the EEG are harmful and should be treated is deeply embedded in current practice. However, if this assertion is not correct and there are other more important determinants of outcomes, then it is critically important that those pathophysiological phenomena are studied, and new treatments developed.

We accept that the recognition of electrographic seizures is important for understanding an overall clinical picture. However, the most critical question should be whether these electrographic phenomena cause brain injury in a way that negatively impacts the outcomes for these children. The evidence supporting this view is lacking and, if anything, there is accumulating evidence that short electrographic seizures, in the absence of electrographic status epilepticus, do not significantly impact the outcomes [4]. There remains controversy about whether electrographic status epilepticus is harmful [2,4,10]. Therefore, the distinction between electrographic seizures and electrographic status epilepticus is important to define. The widely accepted definition of an electrographic seizure is an abnormal, paroxysmal EEG change from the baseline that lasts longer than 10 s with a plausible electrographic field as well as evolution in the frequency and morphology that is not associated with any obvious clinical correlate. Electrographic status epilepticus has been commonly defined as EEG changes lasting >30 min or recurrent electrographic seizures totaling >30 min in 1 h. The average length of an electrographic seizure is less than 1 min and most electrographic seizures are brief and self-resolving [5]. In this review, we argue that the evidence that seizures are harmful deserves a critical evaluation. We will initially address the relationship between convulsive status epilepticus and adverse outcomes as this is considered the situation in which brain injury is most likely to occur.

Status Epilepticus and Brain Injury: There is a long-standing hypothesis that status epilepticus leads to neuronal death and makes a significant contribution to adverse outcomes [11]. The primary source of this hypothesis derives from animal models in which status epilepticus is chemically or electrically induced [12,13,14,15]. Many of these models display evidence for brain injury, particularly to the hippocampus [12,16]. The potential mechanisms that underlie brain injury include excitotoxicity [17,18] and inflammation [19,20,21]. Administration of anti-excitotoxicity agents such as MK-801 are effective at preventing brain injury and epileptogenesis if administered prior to the induction of status epilepticus [22]. Administration of MK-801 at the end of status epilepticus still reduces brain injury, but not epileptogenesis [23]. Despite these results, the clinical use of ketamine (another anti-excitotoxicity agent) has not become widespread. The activation of inflammatory processes has also been hypothesized to be a mechanism underlying brain injury in status epilepticus [19,20,21,24,25,26,27]. In animal models, Cox-2 inhibitors [26], erythropoietin [28,29] and corticosteroids [30] have been tested as neuroprotective agents. There is evidence both in support and against the use of these agents. Recently, a clinical trial of erythropoietin in hypoxic-ischemic encephalopathy showed no benefit beyond the benefit of cooling [31]. Therefore, even if these mechanisms are important for the determination of outcomes, the preclinical evidence that treating on the basis of these mechanisms is weak. Therefore, the advice has been to treat status epilepticus directly so that these injurious mechanisms do not come into play.

The suggestion that the injury is a direct consequence of seizures has been extremely influential in guiding clinical concepts about the investigation and treatment of status epilepticus in humans. However, there are other possible explanations for the findings and there should be caution with respect to translating the animal data to the human situation. It has been extensively shown in animal models that there are many strategies for inducing status epilepticus that are associated with subsequent brain injury. However, there are also chemical models of status epilepticus that do not display subsequent brain injury (e.g., the pentylenetetrazole model) [32]. Interestingly, this was the case even when the seizure manifestations did not significantly differ from those observed in other models that are associated with injury. This raises the issue of why there is no consistent injury across all models and may suggest that the mechanism of inducing the seizure is important in determining the outcome from the seizure. In terms of translatability, these models are best considered as models of status epilepticus in the context of an acute brain insult in a person with a previously normal brain. They may not be good models for studying the consequences of status epilepticus in people with pre-existing brain diseases that predispose to seizures. This obviously includes patients with epilepsy, but may also apply to people with genetic and metabolic disorders. This idea has not been tested for status epilepticus. However, there is no additional negative impact on outcomes in an animal model of the malformation of cortical development after the induction of frequent seizures in the neonatal period [33]. Thus, the underlying etiology is the critical predictor of outcome and not any seizure related phenomena. The severity and timing of acute neurological insults such as hypoxia and infections will influence the severity of downstream brain injury and also influence whether the insult will lead to status epilepticus.

Studies in humans with status epilepticus do not consistently show relationships between any seizure characteristics such as duration or focality and the outcomes [34,35,36]. If the seizure is a fundamental contributor to outcome, then such a correlation would be expected, given the evidence above that excitotoxicity and inflammation may lead to brain injury with status epilepticus but not with short seizures. The lack of correlation strongly suggests that even if status epilepticus per se contributes to brain injury, there must be other factors such as the etiology of status epilepticus that are also important. The most common form of status epilepticus in children is febrile status epilepticus, which occurs in children that are normally developing and without a known underlying neurological disease [37,38]. The evaluation of outcomes in this group of children therefore provides the best human evidence of whether status epilepticus causes brain injury in a situation in which etiology is unlikely to be making a major contribution. There are two major studies evaluating the outcomes from febrile status epilepticus [37,39,40,41]. Both show that there is an uncommon association between febrile status epilepticus and abnormalities identified in the hippocampus within 2 days of termination of the event. A 10 year follow up of the London Cohort revealed excellent outcomes with very low incidence of new neurological (including epilepsy) or cognitive impairments and no correlation between seizure duration and those outcomes [34]. Thus, the evidence for the seizure per se being injurious is weak. Against that background, it is important to note that children with pre-existing neurological impairments also do not seem to show long-term worsening of their impairments, although this is more difficult to measure [34]. These latter children certainly have worse neurological outcomes than children with febrile status epilepticus, but this is a function of the underlying etiology. It is also likely that adverse outcomes in children with TBI, meningitis, metabolic disorders, etc. that have seizures is also heavily a function of the underlying etiology. Therefore, there is reason to reconsider the negative independent impacts of status epilepticus and by logical extension, the treatments. The observations above relate to convulsive status epilepticus, considered the most dangerous seizure type. This suggests that ‘lesser’ seizure types such as electrographic seizures are likely to be less harmful, and therefore the current approaches to this phenomenon also deserve reevaluation.

Treatment of Electrographic Seizures: The current recommendations are that electrographic seizures in critically ill children should be treated with antiseizure medications [6]. This advice deserves critical evaluation given the increasing recognition that short electrographic seizures are not associated with adverse outcomes [4]. However, it is also worth considering whether the subset of children with electrographic status epilepticus should be treated.

In a recent large prospective study, electrographic status epilepticus was associated with worse neurobehavioral outcome after adjusting for variables such as age, acute encephalopathy category, encephalopathy severity (initial EEG background category and comatose state at CEEG initiation), and critical illness severity [4]. Electrographic status epilepticus did not predict mortality. This supports the idea that status epilepticus is harmful, and that treatment could improve the outcomes. However, several other variables such as EEG background activity, the presence of coma at presentation, and the presence of prior epileptic seizures were also strong predictors of outcome including mortality. Importantly, the presence of previous seizures predicted a good outcome. In all circumstances, the odds ratio for the prediction of adverse outcomes was greater for the non-seizure related EEG abnormalities than the odds ratio for the association between electrographic status epilepticus and adverse outcome. Statistically correcting for these other variables does not make them clinically irrelevant, but rather, should highlight their importance.

This idea that non-seizure EEG abnormalities are better predictors of outcome is supported by other studies showing that the more ‘malignant’ background EEG patterns with or without interictal discharges such as burst suppression, diffuse attenuation, or discontinuous features, and poor sleep spindles are associated with worse outcomes. These backgrounds are also associated with higher seizure burdens [10] compared to milder features that are associated with better outcomes [42]. This suggests that it is the degree of injury, rather than the presence of electrographic seizures that is a major contributor to the developmental outcomes. When looking at neurological outcomes in post cardiac arrest children, Smith et al. found that while the presence of clinical status epilepticus was associated with increased risk of death and unfavorable prognosis, isolated clinical seizures did not have the same association with neurological outcomes [43]. When further dissected into the presence of electrographic seizures in post cardiac arrest patients, they found no difference in the outcomes in those with electrographic seizures and those without. The more important diagnostic indicator of prognosis was background abnormality, a marker of the severity of brain injury. Importantly, there was again an interaction between the severity of background abnormalities and presence of electrographic seizures. Those with more malignant background abnormalities (unreactive, discontinuous, poor sleep transients) were associated with a higher electrographic seizure burden [4,43]. This suggests that other factors play more into the worse neurological outcomes than the electrographic seizures themselves, and that the seizures are more a marker of injury than a modifiable risk factor. Future research that attempts to understand the other factors and define treatment strategies has much potential for improving the outcomes.

These ideas on the association between adverse outcomes and electrographic seizures versus clinical seizures alone extend to the neonatal period. There are many studies that have shown a correlation between electrographic seizures and outcomes [44,45,45,46]. In a recent study, full-term and near-term neonates with clinical seizures alone or clinical seizures plus electrographic seizures, regardless of etiology, were recruited [47]. At 2-year follow-up, there was no difference in the mortality and neurodevelopmental disabilities between the groups [47]. It should be noted that this study utilized amplitude-integrated EEG, thus seizures were not validated with conventional EEG. Nevertheless, the presence of electrographic seizures did not predict adverse outcome, and thus supports the view that etiology is critical. The heterogenous etiology in the recruited neonates made it difficult to precisely define the complex relationship between the cause of brain injury and neonatal seizures. However, in the neonate, as with older children, there may be a relationship between high seizure burden and negative short-term and long-term neurological outcomes including prolonged hospitalization, abnormal neurological exam on discharge, microcephaly, cerebral palsy, and failure to thrive [44,46,48]. Thus, neonatal status epilepticus, and not recurrent seizures, has been shown to be a risk factor for neurodevelopmental disabilities and postnatal epilepsy at 24 months [49]. The question of whether the high seizure burden causes or simply correlates with outcomes remains unanswered.

Non-seizure related factors that could be important include the timing of brain injury and/or dysfunction relative to the antepartum, intrapartum, and postpartum dynamics that can impact brain function. There is increasing recognition that there is an intricate association between maternal and fetal factors that influences brain development and its manifestations, which include neonatal seizures [44]. Moving the focus of neonatal brain injury from aggressive treatment of seizures to understanding the environment–mother–fetus interactions could ultimately have a far greater impact on the outcomes from neonatal brain injury than the treatment of seizures. The nature and timing of the disruption to developing neural networks in an environment–mother–fetus frame could also have important implications for the children that have electrographic seizures in later childhood and perhaps even adulthood. It remains unknown whether later outcomes differ as a function of the cause of early neurological insult or not. Altogether, it is likely that there is little causative association between neonatal seizures and poor outcome; instead, it is a multifaceted phenomenon that questions the efficacy of the concrete approach to abolish all seizures.

Neurologists and intensivists have been aggressively treating seizures and electrographic abnormalities in the EEG for many years. There have been improvements in the quality of EEG monitoring, decreased time to EEG in ICU settings, more rapid recognition of seizures, and increasing antiseizure medication treatment options [9,50]. However, the clinical validity of these efforts with respect to improvement in the overall outcomes remains uncertain. It is interesting that the literature is awash with papers showing a relationship between status epilepticus and outcomes, but there are very few papers addressing whether there are improved outcomes with treatment. It is difficult to know whether this is a function of publication bias in which negative studies are not reported or whether the studies have not yet been rigorously performed. Because of the existing bias toward acceptance that status epilepticus is harmful, a traditional randomized controlled trial of treatment versus no treatment is unlikely to be completed. However, there has been an excellent study from Switzerland carried out in adults with electrographic, but not clinical seizures, in the intensive care setting [51]. The authors randomized patients by having continuous EEG monitoring for 36 to 48 h, or two routine EEGs over the same time period [51]. The patients that were continuously monitored received more antiseizure medications, but there was no difference in the mortality or cerebral performance at 6 months follow up. This finding is supportive of the underlying etiology of brain injury being more significant with regard to poor neurological outcome and that the seizures are merely a biomarker of brain injury rather than a modifiable risk factor. Thus, there is reasonably strong evidence that current consensus statements regarding the emergent need for continuous video EEG monitoring in critically-ill patients are based on the correlation rather than causation of seizure burden with acute brain injuries and poor outcomes. Perhaps it is time to revisit these statements in light of new evidence.

The question of whether treatment of seizures worsens certain outcomes also deserves consideration. Aggressive treatment of seizures could not only prolong mechanical ventilation in critically-ill patients, but also prolong sedation, increase the days of suboptimal nutrition, prolong hospital length of stays, and increase rehabilitation needs post-acute management. Unfortunately, most of these potential impacts have not been investigated. The one area that has been studied is the length of stay in the intensive care unit. In a very large study of 16,928 adults with non-traumatic subdural hematomas, the length of intensive care stay increased from 3.36 days to 9.36 days in patients with seizures compared to those without seizures [52]. The size of studies with children is smaller, but there is evidence that the duration of PICU stay in children with electrographic seizures is longer than that in critically ill children without seizures, and this duration increases further with electrographic status epilepticus [2,4]. It remains uncertain whether the increased duration of stay is a function of the underlying brain insult or aggressive use of antiseizure medications. Nevertheless, children with electrographic status epilepticus are exposed to a much higher number and dose of medications, many of which are sedating [6]. Almost half of children with electrographic status epilepticus receive at least four antiepileptic agents. Pentobarbital infusions, midazolam infusions, or isoflurane were used in 25% of those with electrographic status epilepticus [6]. It should be noted that even those children with electrographic seizures and no episodes of status epilepticus had prolonged PICU stays compared to critically ill children with no seizures. This may suggest that even the more traditional medications including lorazepam, levetiracetam, phenytoin, or sodium valproate are having a negative impact on outcomes. Thus, in cases of low seizure burden, if electrographic seizures play a limited role in contributing to worse neurological outcomes, then the aggressive management of electrographic seizures may expose certain cohorts of patients to increased risk associated with intensive care admission [53,54]. Ultimately, it raises the question of what the treatment regimen is doing “to” the child versus “for” the child. Many times, limited intervention could have a much more meaningful impact on trajectory through PICU and the ultimate outcomes if one has the patience to assess the patients’ entire clinical picture, rather than narrowly targeting electrographic seizures. Treatment strategies tailored toward interventions when seizure burden breaches above a certain threshold such as >30 min of seizure activity/hour where risks for negative neurological outcome increases are more justifiable, although also on a weak conceptual footing.

We recognize that practice is unlikely to change rapidly and acknowledge that cEEG will continue to be requested in the PICU. cEEG monitoring requires substantial hospital resources and therefore should target the cohort of critically ill children who are most at risk for electrographic status epilepticus, allowing for the more optimal use of limited resources. In a prospective observational study by Abend et al., seizures were identified in 87% of critically-ill children within the first 24 h of cEEG monitoring [55]. Therefore, we propose that if no evidence of status epilepticus is identified within the first day of monitoring, then no further monitoring is required. It is also important that excessive treatment is not given to children with short electrographic seizures simply because they are being monitored. Based on the current data of when an increased risk of neurological decline occurs with regard to electrographic seizure burden, the threshold to treat can be set at electrographic status epilepticus or seizure burden >30 min/h. The presence of seizures below this threshold does not impact the neurological outcome in either the short- or long-term, and thus aggressive treatment could possibly lead to iatrogenic morbidities including prolonged ventilation, prolonged sedation, and thus a delay in rehabilitation intervention for recovery.

In summary, we propose that aggressive management to cease all electrographic seizures may have little value and carries potential risk. Rather, we suggest that treatment should be tailored to the underlying etiology of the neurological injury using the EEG background as a guide to establish a threshold above which a negative neurological outcome is more probable. The approach to ICU cEEG monitoring in critically ill children should be based on what will ultimately improve their neurological outcomes in the long-term rather than short-term gratification with the cessation of electrographic seizures. After the evaluation of the current data, we recommend re-examining our current consensus practice of emergent EEGs and the aggressive treatment of isolated electrographic seizures. We suggest limiting unnecessary interventions if the ultimate outcome is unchanged.

It is clear that EEG in the PICU setting is useful for prognostication in terms of both abnormal background activities and the presence of electrographic status epilepticus. There is mounting evidence that the treatment of short electrographic seizures does not alter outcomes and may have adverse effects such as a prolongation of PICU stay. The remaining question of whether treatment of electrographic status epilepticus alters outcomes remains uncertain, and therefore the treatment of electrographic status epilepticus remains valid, albeit in the knowledge that we may be having limited meaningful clinical impact. Future studies focusing on improving outcomes should include metrics that address the impact of etiology in relation to the treatment of electrographic status epilepticus.

## Data Availability

No new data were created or analyzed in this study. Data sharing is not applicable to this article.
